# Nephron sparing surgery: Is here to stay

**DOI:** 10.4103/0970-1591.30261

**Published:** 2007

**Authors:** Ganesh Gopalakrishnan

**Affiliations:** Department of Urology, Christian Medical College, Vellore - 632004, India. E-mail: ganeshgopalakrishnan@yahoo.com

While radical nephrectomy is still the gold standard of treatment for renal cell carcinoma, its role is now being seriously challenged. As one surveys world literature, it has become evident that numerous articles have shown the long-term safety both surgically and oncologically of nephron sparing surgery (NSS).

What does one mean by the term “Nephron Sparing Surgery”. Loosely it could be used to include any surgical procedure that preserves nephrons. This includes a whole list of procedures for both benign and congenital anomalies.

I believe we need to restrict the use of the term NSS for the treatment of renal cell carcinoma when conventionally we have all been doing a radical nephrectomy. For example, removal of a non-functioning duplex moiety should not be classified as NSS.

There are two articles in this issue of the journal that report single center experience of NSS.[1] In one of them the strict definition as I had stated has not been followed. While the place of NSS is now established, as urologists we need to remember two issues. First, is a commitment to ensure adequate and accurate surveillance of these patients and secondly if adequate follow-up cannot be guaranteed, a problem in our country, then elective NSS is better not performed especially in the over 60 years age group.

Additionally in our country, when treatment and follow-up costs are invariably borne by the patient, it might seem prudent to offer a radical nephrectomy for the “elective NSS” at least in the older population. In our enthusiasm to perform NSS we should also be focused on local patient demographics. Certainly in the younger patient NSS is a strong option. One cannot predict in the absence of a strong family history if a younger person will develop hypertension and/or diabetes in later life. We need to remember that soon the Indian subcontinent will have the largest number of diabetes in the world (International Diabetes Federation Report).[2] At this time a maximal amount of nephron mass is important to reduce the effects of target organ damage.

The oncological safety of NSS for a < 4 cms renal neoplasm is now fairly well established in the category of elective NSS.[3–6] The five-year cancer specific survival of a 97.8% for tumor < 4 cm treated by partial nephrectomy compares with a similar survival when treated with radical nephrectomy. The articles in this issue have addressed this modality of treatment with good short-term outcome. I believe we need longer follow-up. The controversy is the role of NSS for tumors between 4–7 cm. These articles do not address this issue. Literature while suggesting the successful use of partial nephrectomy for such lesions, is limited by small numbers and short term follow-up.[7] In the subgroup univariate and multivariate analysis, the most important predictor of outcome was tumor size. In my opinion, measurement of tumor size by imaging or at surgery and by the pathologist on the specimen are quite in variance with one another and could make a difference at the upper end of the spectrum. Similarly some large tumors could also be undersized. Unfortunately these corrections are not taken into our analysis.

At this time, I believe that we need to remember the facts while offering treatment for patients with renal cell carcinoma. Radical nephrectomy is still the gold standard. For a 4 cm tumor elective partial nephrectomy matches currently the survival figures for radical nephrectomy.[3] In the younger patient this would make sense as the genetic risk of another lesion in this or the opposite kidney is a serious threat. In the older patient it probably does not make a difference. For the large tumor (4–7 cm), I believe the jury is still out and we need to exercise great caution while offering NSS to this group on an elective basis.
